# Supplementary material for: Atlantic Water warming increases melt below Northeast Greenland’s last floating ice tongue
Source: Nat Commun. 2024 Feb 20;15:1336. doi: 10.1038/s41467-024-45650-z (PMC10879102; doi:10.1038/s41467-024-45650-z)
Supplement: Supplementary file 1 — Supplementary Information [file 41467_2024_45650_MOESM1_ESM.pdf]

Supplementary Information: Atlantic Water warming increases melt below Northeast Greenland's last floating ice tongue

Claudia Wekerle[1,*], Rebecca McPherson[1], Wilken-Jon von Appen[1], Qiang Wang[1], Ralph Timmermann[1], Patrick Scholz[1], Sergey Danilov[1,2], Qi Shu[3] and Torsten Kanzow[1,4]

[1]Alfred Wegener Institute, Helmholtz Centre for Polar and Marine Research, Bremerhaven, Germany
[2]Department of Mathematics and Logistics, Jacobs University, Bremen, Germany
[3]First Institute of Oceanography, Ministry of Natural Resources, Qingdao, China
[4]University of Bremen, Bremen, Germany
[*]Corresponding author: Claudia Wekerle (claudia.wekerle@awi.de)

Contents:

This supplementary material contains additional figures in support of the findings described in the main manuscript:

Supplementary Figure 1: Model setup and topography
Supplementary Figure 2: Basal melt rate
Supplementary Figure 3: Exchange flow at the calving front during times of low/high basal melt
Supplementary Figure 4: Impact of basal friction

Supplementary References

[1] Wilson, N., Straneo, F. & Heimbach, P. Satellite-derived submarine melt rates and mass balance (2011–2015) for Greenland's largest remaining ice tongues. *The Cryosphere* **11**, 2773–2782 (2017).

[2] Schaffer, J. *et al.* An update to Greenland and Antarctic ice sheet topography, cavity geometry, and global bathymetry (RTopo-2.0.4) (2019).

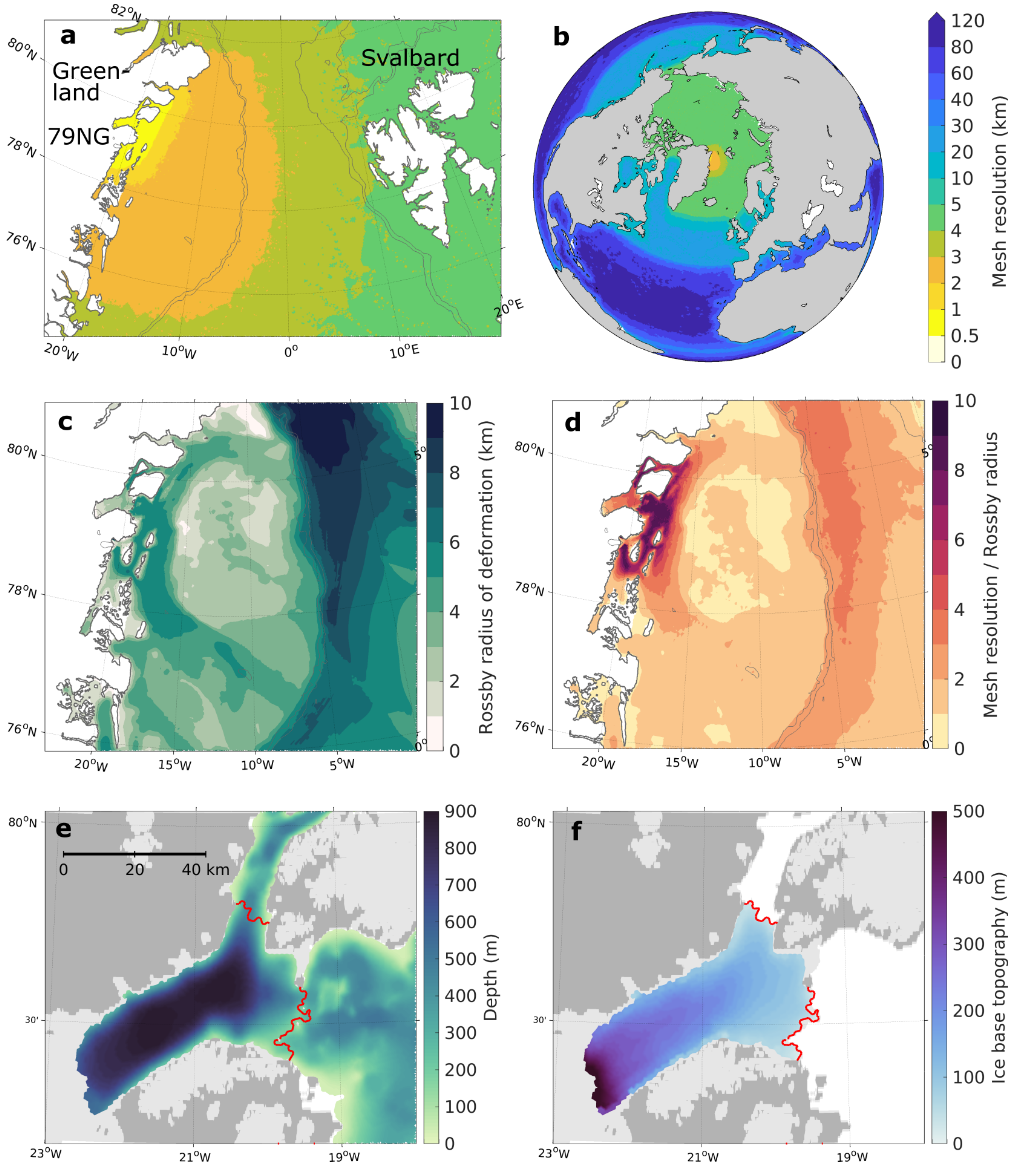

Supplementary Figure 1: **Model setup and topography.** Mesh resolution in **a** Fram Strait and on the Northeast Greenland continental shelf and **b** global view. Around the NEG glaciers, mesh resolution is set to 700 m. Note the non-linear colour scale. Grey contours indicate the 1000 m and 2000 m isobaths. **c** Rossby radius of deformation computed from the simulated long-term mean density field and **d** ratio of mesh resolution to Rossby radius. **e** Water depth and **f** ice base topography in the model based on data from RTopo-2.0.4 [2]. The red line shows the calving front. The grey and light grey background shows bare land and grounded ice, respectively.

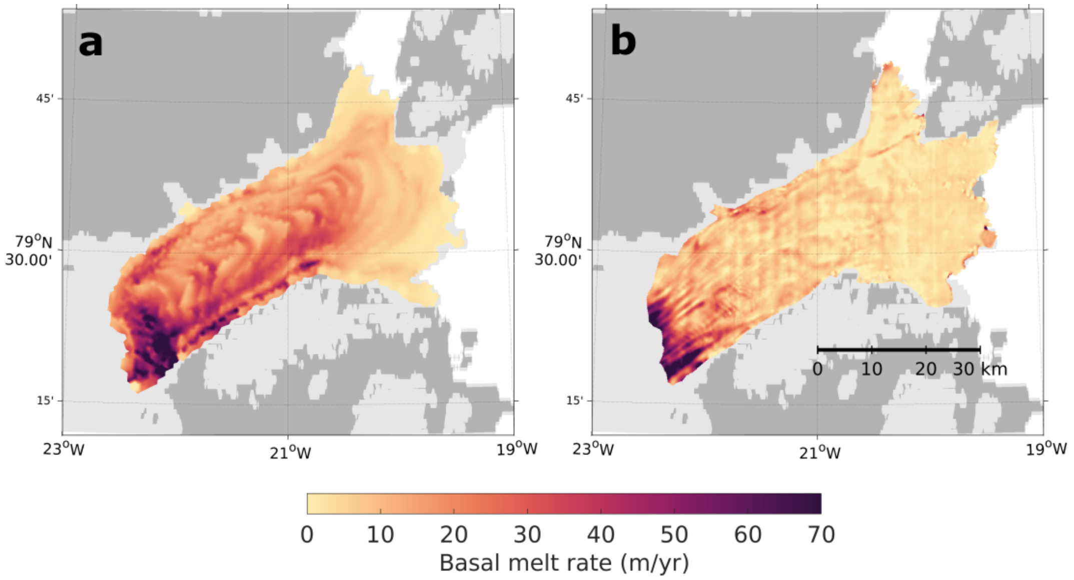

Supplementary Figure 2: **Basal melt rate** from **a** FESOM2.1 simulation (average over 2011–2015) and **b** satellite altimetry (redrawn from [1]). The grey and light grey background shows bare land and grounded ice, respectively.

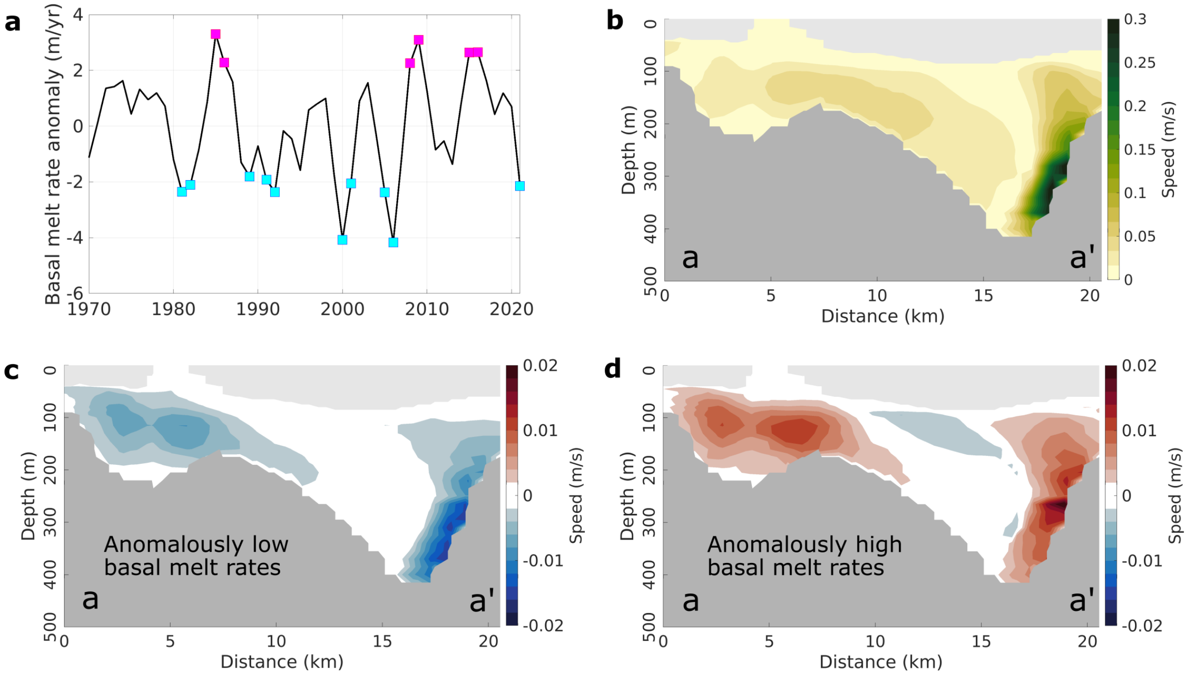

Supplementary Figure 3: **Exchange flow at the calving front during times of low/high basal melt. a** Detrended time series of anomalous annual mean basal melt rate. Years with values above/below one standard deviation are indicated by magenta/cyan squares. **b** Long-term-mean cross-sectional speed at the calving front (section a–a' in Figure 2b). **c,d** Anomalous cross-sectional speed at the calving front (section a–a') during years of low/high basal melt rate. In years of low/high basal melt rates, the inflow and outflow is weaker/enhanced. Velocity fields were detrended prior to computing composites.

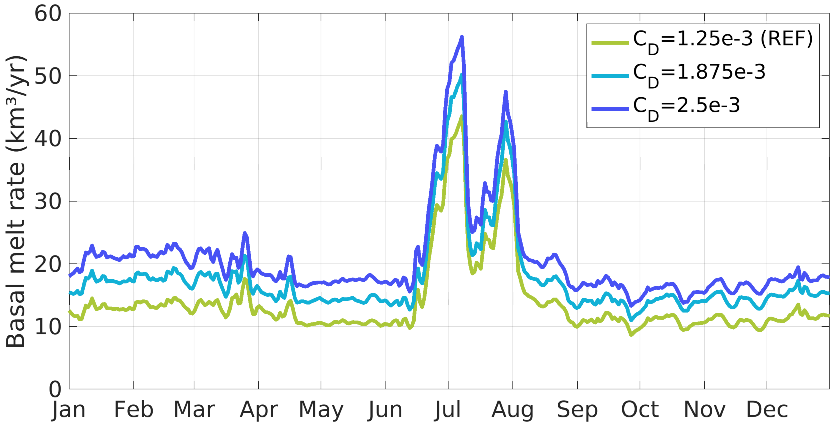

Supplementary Figure 4: **Impact of basal friction.** Time series of daily mean basal melt rate in the year 2000 in experiment REF (green line, using a drag coefficient of $C_D = 1.25 \cdot 10^{-3}$), and experiments with higher drag coefficient ($C_D = 1.875 \cdot 10^{-3}$, turquoise line and $C_D = 2.5 \cdot 10^{-3}$, blue line).
